# Effect of Noise and Music on Neurotransmitters in the Amygdala: The Role Auditory Stimuli Play in Emotion Regulation

**DOI:** 10.3390/metabo13080928

**Published:** 2023-08-08

**Authors:** Haoyang Nian, Susu Ding, Yanru Feng, Honggui Liu, Jianhong Li, Xiang Li, Runxiang Zhang, Jun Bao

**Affiliations:** 1 College of Animal Science and Technology, Northeast Agricultural University, Mucai Street No. 59, Harbin 150030, China; 2 Key Laboratory of Swine Facilities Engineering, Ministry of Agriculture and Rural Affairs, Changjiang Road No. 600, Harbin 150030, China; 3 College of Life Science, Northeast Agricultural University, Mucai Street No. 59, Harbin 150030, China

**Keywords:** neuroinflammation, metabolomics, piglets, amygdala, noise, GABA

## Abstract

Stress caused by noise is becoming widespread globally. Noise may lead to deafness, endocrine disorders, neurological diseases, and a decline in mental health. The mechanism behind noise-induced neurodevelopmental abnormalities is unclear, but apoptosis and pro-inflammatory signals may play an important role. In this study, weaned piglets were used as a model to explore noise-induced neurodevelopmental abnormalities. We hypothesized that long-term noise exposure would induce anxiety and cause acute stress, exhibited by alterations in neurotransmission in the amygdala. A total of 72 hybrid piglets (Large White × Duroc × Min Pig) were randomly divided into three groups, including noise (exposed to mechanical noise, 80–85 dB), control (blank, exposed to natural background sound, <40 dB), and music (positive control, exposed to Mozart K.448, 60–70 dB) groups. The piglets were exposed to 6 h of auditory noise daily (10:00–16:00) for 28 days. Compared with the control group, piglets exposed to noise showed more aggressive behavior. The expression of Caspase3, Caspase9, Bax, NF-κB (p56), TLR4, MYD88, I κ B α, IL-1 β, TNF-α, and IL-12RB2 was significantly upregulated in the amygdala, while the expression of Nrf2, HO-1, CAT, and SOD was downregulated in piglets in the noise group. Cell death occurred, and numerous inflammatory cells accumulated in the amygdala of piglets in the noise group. Targeted metabolomics showed that the content of inhibitory neurotransmitter GABA was higher in the amygdala of piglets in the noise group. Compared with the noise group, piglets in the music group displayed more positive emotion-related behaviors. Compared with the noise group, the expression of genes related to apoptosis, inflammation, and oxidative damage was lower in the music group. Cells of the amygdala in the music group were also of normal morphology. Our results show that noise-induced stress causes apoptosis and neuroinflammation in the amygdala and induces anxiety during the early neonatal neural development of piglets. In contrast, to some extent, music alleviates noise-induced anxiety.

## 1. Introduction

Noise exists widely in the environment, and noise exposure may cause loss of hearing, insomnia [1], cardiovascular diseases [2], and effects on mental health [3]. Noise causes several other negative effects. For example, it can lead to apoptosis [4,5] and oxidative damage [6]. In addition, noise can also alter calcium levels, which causes cell death [7]. Noise, an environmental stressor, has the potential to cause cell death and apoptosis.

Exposure to noise can affect mental health, leading to the occurrence of negative emotions. Responses may be regulated directly or indirectly in the amygdala [8]. The amygdala plays an important role in the processing and coding of negative stimuli. The pathway in the amygdala guards against negative stimuli, which mediates reflexive behavioral responses [9,10]. Pain [11], anxiety [12], and a series of complex neural activities are often regulated by amygdala neurons [13]. In addition to coding information related to negative emotions, the amygdala is highly sensitive to auditory stimuli. Neuroimaging and other techniques have revealed the function of the amygdala in perceptual audio coding. Specifically, the amygdala participates in auditory fear conditioning [14] and the perception of vocally expressed emotions [15], suggesting that the amygdala may have an independent regulation mechanism for auditory-related negative stimuli.

Generally, changes in the content of neurotransmitters may be a strategy to cope with external stress. Many studies have shown that noise stress affects the metabolism of neurotransmitters. For instance, chronic noise stress alters the excitability of glutamic acid by disrupting the balance between the GABA-inhibitory and glutamate-excitatory systems [16,17]. Glutamic acid is the main neurotransmitter that regulates rapid excitatory synaptic responses, while GABA, in contrast, is an important inhibitory neurotransmitter. Therefore, imbalances in these two main neurotransmitters directly disrupt endocrine activity and cause behavioral abnormalities. A study showed that noise stress exposure significantly increased the levels of monoamine neurotransmitters (e.g., dopamine, noradrenaline, etc.) [18], implying that noise stress disrupts the expression of neurotransmitters. However, some studies have demonstrated that noise has no effect on the metabolism of neurotransmitters [19]. Thus, whether noise exposure could dysregulate the secretion and metabolism of neurotransmitters in piglets needs further research. The amygdala is the processing center for negative auditory signals and emotions; therefore, the neurotransmitter metabolism after exposure to noise stress deserves in-depth study.

The amygdala, a highly differentiated part of the brain, is essential in regulating negative stimuli. Herein, weaned piglets were exposed to noise stress to explore whether noise stress causes the apoptosis of amygdala cells, inflammation, and oxidative damage. The ways in which the changes induced by noise stress affected the growth and development of amygdala neurons were also investigated. Finally, we investigated the interaction between the metabolism of neurotransmitters and behavior patterns. Piglets have anatomical structures and physiology similar to those of human infants [20,21]. Therefore, the findings of this study could provide practical evidence of how auditory stimulation mediates neuroinflammation, emotional regulation, and neurotransmitter metabolism, all important in promoting advances in comparative medicine.

## 2. Materials and Methods

### 2.1. Ethics Statement

The experimental protocol was approved by the Animal Ethics Committee of the Northeast Agricultural University (project number: 31972606). The sampling procedures were performed in accordance with the Guidelines on Ethical Treatment of Experimental Animals (2006) (No. 398) of the Ministry of Science and Technology, China.

### 2.2. Animals and Management

A total of 72 hybrid piglets (Large White × Duroc × Min Pig) with a weight of 8.09 (±2.01) kg and an age of 40 (±3) days were selected from the university experimental farm in Harbin, Heilongjiang, China. These piglets were given iron supplements (Yuanda Animal Health Products Co., Ltd., Jinan, China) on the 3rd day after birth, castrated on the 21st day, and weaned on the 30th (±3) day. A commercial creep feed was fed on the 14th day, the piglets were given free access to water and feed, and they were kept in 12 equal-sized pens (6 piglets in each pen, including 3 males and 3 females, SF1) [22] and fed at 8:00–17:00 daily with an average amount of feed of 0.87 kg/d/piglet before the 28th day of the test. The feed supplied to the piglets contained 11.87 MJ/kg in energy, and the crude protein, crude fiber, and lysine content were 16.5%, 5%, and 1.2%, respectively. The daily temperature and humidity of the testing room were 20–25 °C and 60–75%, respectively; during the experimental period, natural ventilation was adopted. Lighting was given at 20l× per day at 8:00–20:00. Piglets’ health was inspected routinely each day.

### 2.3. Experimental Procedure

The piglets were randomly divided into 3 groups, including noise, control (perform as blank control), and music (perform as positive control) groups, and raised in 3 separate equal-sized rooms (3 (W) × 3.8 (L) m^2^) with 4 replicate pens (l.8 m length × 1.2 m width × 1 m height, SF1, [22] equipped with a waterer, a trough, and a slatted floor. There were 6 piglets in each pen, providing a stocking density of 0.36 m^2^/piglet. Noise (80–85 dB) comprised a recording of mechanical fan noise, as well as other background sounds from an animal house; the sound in the control group was the natural background sound of the experimental rooms, which was approximately 40 dB. Mozart’s sonata K.448 (60–70 dB) was adopted in the music group. Speakers with right and left channels were set in the middle of the ceiling, and the sound volume of each pen was the same, measured by a digital sound level meter (AS804, SMART SENSOR Co., Ltd., Hong Kong, China). Auditory treatment was played for 6 h (10:00–16:00) daily in a regular loop. The experiment lasted for 28 days.

### 2.4. Serum Sampling and Measurement of Oxidative Stress

On day 28, a piglet was randomly selected from each pen and then euthanized to collect blood samples (5 mL). After standing for 4 h, the supernatant was collected and centrifuged at 3000 rpm for 10 min at room temperature. Serum was collected and transformed into a 1.5 mL microcentrifuge tube and stored at −20 °C. The activity of catalase (CAT) and superoxide dismutase (SOD) was measured using respective kits purchased from Nanjing Jiancheng Biotechnology Co., Ltd. (Nanjing, China), according to the manufacturer’s instructions. The intra-assay coefficient of variation was less than 10%, the inter-assay coefficient of variation was below 12%, and the limit of detection was 0.5 U/mL. The absorbance (OD) value of the sample was determined by an enzyme labeling instrument, and the standard curve was fitted and calculated according to the concentration and OD value of the standard sample.

### 2.5. Tissue Sampling and Measurement of Cytokines and Oxidative Stress

On day 28 of the experiment, a piglet was randomly selected from each pen, euthanized, and tissue samples collected. The amygdalae were collected, flash-frozen in liquid nitrogen, and then stored at −80 °C. The concentrations of interleukin-10 (IL-10) (the minimum detection limit was 5 ng/L) and interleukin-12 (IL-12) (the minimum detection limit was 5 ng/L) were measured using commercially available ELISA kits (Xinle Biotechnology Co., Ltd., Shanghai, China), according to the manufacturer’s instructions. The activities of CAT and SOD in the amygdala were also measured, as described in Section 2.5.

The intra-assay coefficient of variation was less than 10%, and the inter-assay coefficient of variation was below 12%. The OD value of the sample was determined by an enzyme labeling instrument, and the standard curve was fitted and calculated according to the concentration and OD value of the standard sample.

### 2.6. Histopathological Studies

Fresh amygdala tissue was soaked in 10% formaldehyde for 24 h, embedded in paraffin, cut into 5 μm sections, stained with hematoxylin and eosin (H&E), dried, and observed under an optical microscope. The slides were digitized with a Pannoramic MIDI scanner (3DHISTECH; Budapest, Hungary), and the micrographs were read using the CaseViewer software (Version 2.4, 3D HISTECH, Budapest, Hungary).

### 2.7. Electron Microscopy

Fresh amygdala tissue was fixed in 2.5% glutaraldehyde phosphate buffer at 4 °C for 2 h. These tissue samples were fixed in 1% osmium tetroxide (OsO4) for 2 h after cleaning with phosphate-buffered saline, dehydrated, soaked, embedded, sectioned, and stained. Finally, cells were observed by transmission electron microscopy (Hitachi, H-7650).

### 2.8. Quantitative Real-Time PCR Analysis

Total RNA was isolated from the amygdala with TRIzol reagent (Invitrogen, Carlsbad, CA, USA). RNA quantity and quality were determined using a micro-spectrophotometer (Eppendorf, Hamburg, Germany). Then, 1 μg of total RNA was applied for reverse transcription with an RT kit (Beyotime, Beijing, China). The primers used in this study are listed in Supplementary Table S1. qRT-PCR was performed on a LightCycler^®^ 480 System (Roche Diagnostics, Mannheim, Germany) with FastStart Universal SYBR Green Master (ROX) (Roche Applied Science, Mannheim, Germany). Expression levels were determined with the 2^−∆∆CT^ method and normalized to β-actin.

### 2.9. Western Blot Analysis

Total protein was extracted from the amygdala with the lysis buffer for Western and IP (Biosharp, Beijing, China) plus protease inhibitor (Beyotime, Beijing, China). The enhanced BCA assay kit (Beyotime, Beijing, China) was used to determine the protein concentration. Total protein was separated on 10% and 12% SDS-PAGE gel and transferred to nitrocellulose membranes. The membranes were blocked with 5% BSA and then incubated with primary antibodies. The membranes were incubated with a horseradish peroxidase (HRP)-conjugated secondary antibody, and the dilution ratios of the primary antibodies used in this experiment are listed in Supplementary Table S2. The protein bands were visualized using an Amersham Imager 600 RGB (General Electric Company, Boston, MA, USA) after being treated with an enhanced chemiluminescence (ECL) reagent (Beyotime, Beijing, China). Obtained bands were quantified using Image J (Version 1.8.0, Rasband, MD, USA).

### 2.10. Neurotransmitter Detection

The UPLC-ESI-MS/MS method was used for the qualitative and quantitative analysis of neurotransmitters. Briefly, samples of amygdala were taken, adding 400 μL precooled (4 °C) methanol–water (*v*/*v* = 4:1, including 0.1% formic acid) and 24 μL precooled succinic acid-d4. The samples were precooled at −20 °C and then ground in a grinder (60 Hz for 2 min). After centrifuging (10 min, 4 °C, 12,000 rpm), the supernatant was discarded. These procedures were repeated once, and then the samples were re-dissolved with 200 μL water (containing 2-chloro-L-phenylalanine) and centrifuged for 5 min (4 °C, 13,000 rpm); 200 μL supernatant was transferred to a brown sample injection vial for LC-MS analysis. The chromatographic conditions (AB ExionLC; AB Sciex, Shanghai, China) included a 0.3 mL/min flow rate, and the mobile phase included A (0.1% formic acid–aqueous solution) and B (0.1% formic acid–methanol). The gradient elution method (Gradient Elution Procedures) was followed: 0 min, A/B (99:1), 1 min, A/B (99:1), 6 min, A/B (5:95), 7 min, A/B (5:95), 7 min, A/B (99:1), and 8 min, A/B (99:1). The conditions of mass spectrometry (AB Sciex Qtrap 6500 V; AB Sciex, Shanghai, China) were as follows: positive ion spray voltage, 5500V; negative ion spray voltage, −4500 V; ion source temperature, 450 °C; column temperature, 40 °C. The chromatographic column was the ACQUITY UPLC HSS PFP (100 mm × 2.1 mm, 1.8 um, Waters, Milford, CT, USA).

### 2.11. Behavioral Observation

The behaviors of the piglets were recorded with IR cameras (Hangzhou Haikang Weishi Digital Technology Co., Ltd., Hangzhou, China) installed on the ceiling. The behavioral observation was performed on the 28th day (10:00–6:00). One piglet was randomly selected from each pen and sampled by focal animal sampling with an interval of 20 s. The state behaviors were recorded and the data were converted into a percentage of the total observation time. The event behaviors were observed using the instantaneous sampling method following the one-zero sampling principle, and the occurrences of event behaviors were recorded as frequencies. The definition of behaviors is shown in Table 1. The field test was carried out by one experimenter, while observation and analysis were conducted by another experimenter to ensure the blindness of the experiments.

### 2.12. Statistical Analysis

The data were processed with the Kolmogorov–Smirnov test for a normal distribution. Normally distributed data were analyzed using a parametric test (one-way ANOVA), while the data that did not conform to a normal distribution were analyzed by a non-parametric test (Kruskal–Wallis test). The results are presented as the mean ± standard error of the mean (SEM). Analysis between groups was determined by multiple comparison tests. *p* < 0.05 was considered a statistically significant difference. All statistical analyses were carried out using SPSS (Version 20.0, SPSS Inc., Chicago, IL, USA).

## 3. Results

### 3.1. Behavioral Responses of Piglets to Noise and Music Exposure

**Figure 1:**
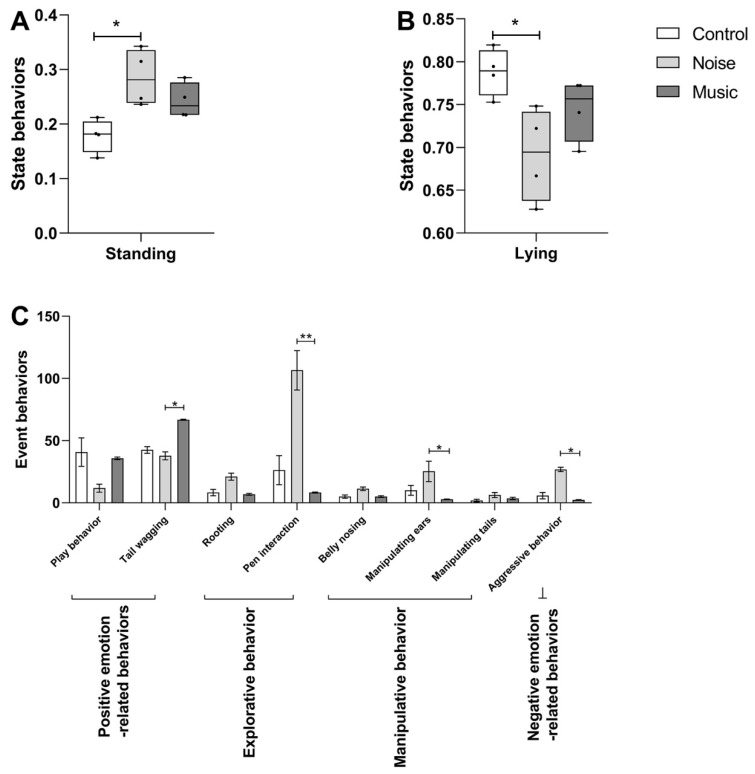
Effect of noise and music exposure on state behaviors of piglets (**A**,**B**) and event behaviors (**C**) among 3 groups. Data are expressed as mean ± SE; data in box-and-whisker graphs are expressed as median +/− quartiles; significant differences are indicated by asterisks (* *p* < 0.05, ** *p* < 0.01; Kruskal–Wallis test, *n* = 4).

The piglets in the noise group were more aroused. Specifically, they spent significantly more time standing than those in the control group (Figure 1A, *p* = 0.0181). However, no significant difference in arousal was observed between the music and the control groups (*p* > 0.05). The lying time of the piglets in the noise group was less than that of piglets in the control group (Figure 1B, *p* = 0.0240), but no significant difference was observed between the noise and the music groups (*p* > 0.05). As shown in Figure 1C, the pen interaction, manipulation of ears, and aggressive behavior in the noise group were more frequent than those in the music group (*p* = 0.0057, *p* = 0.0153, *p* = 0.0171). The positive emotion-related behaviors were more frequent in the music group than in the noise group (*p* = 0.0324). Both the explorative behaviors and the manipulative behaviors were more frequent in the noise group than in the control group, albeit statistically insignificant.

### 3.2. Changes in the Levels of Neurotransmitters in the Amygdala

**Figure 2:**
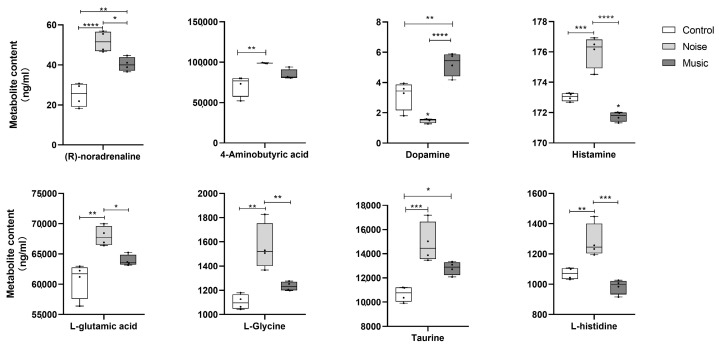
The effects of exposure of piglets to noise and music stimuli on the changes in neurotransmitter concentrations in the amygdala. Data are expressed as mean ± SE; data in box-and-whisker graphs are expressed as median +/− quartiles; significant differences are indicated by asterisks (* *p* < 0.05, ** *p* < 0.01, *** *p* < 0.001, **** *p <* 0.0001 one-way ANOVA, *n* = 4).

The changes in the concentrations of neurotransmitters caused by noise and music are shown in Figure 2. Results showed that noise and music dysregulated the secretion of noradrenaline, GABA, dopamine, histamine, glutamic acid, glycine, taurine, and histidine. Compared with the control group, the concentrations of noradrenaline (*p* < 0.0001), GABA (*p* = 0.0035), histamine (*p* = 0.0003), glutamic acid (*p* = 0.0018), glycine (*p* = 0.0012), taurine (*p* = 0.0010), and histidine (*p* = 0.0073) were higher in the noise group, while the concentration of dopamine (*p* = 0.0210) was lower. The concentrations of noradrenaline (*p* = 0.0051), dopamine (*p* = 0.0065), and taurine (*p* = 0.0493) were higher in the music group than in the control group, while the concentration of histamine (*p* = 0.0500) was lower in the music group than in the control group. Compared with the music group, the concentrations of noradrenaline (*p* = 0.0268), histamine (*p* < 0.0001), glutamic acid (*p* = 0.0487), glycine (*p* = 0.0099), and histidine (*p* = 0.0008) in the noise group were higher, while the concentration of dopamine (*p* < 0.0001) was lower.

### 3.3. Noise Exposure Activated the Apoptosis Pathway in the Amygdala

**Figure 3:**
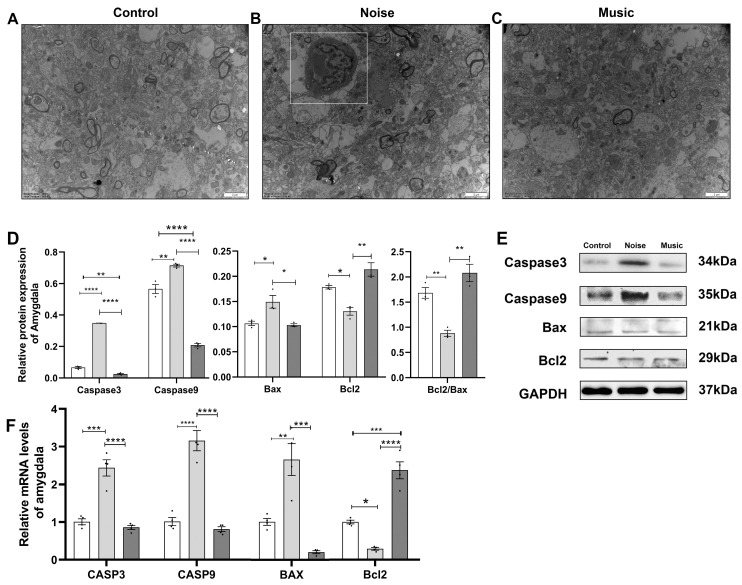
The effect of noise and music exposure on apoptosis. (**A**,**C**) show the ultrastructure of the piglets’ amygdala in the control and music group after treatment; the two groups exhibited normal morphology and no ultrastructural changes appeared. (**B**) shows the ultrastructure of the amygdala in the noise group; the cytoplasm became darker, chromatin in the outer nuclear layer gathered with an uneven distribution, and the death of cells occurred. (**D**) shows the apoptosis levels in the amygdala. The effects of noise exposure on protein levels (**D**,**E**) and mRNA levels (**F**) of apoptosis-related genes in the amygdala. GAPDH was selected as the reference. Data are expressed as mean ± SE; significant differences are indicated by asterisks (* *p* < 0.05, ** *p* < 0.01, *** *p* < 0.001, **** *p <* 0.0001; one-way ANOVA, *n* = 4).

The ultrastructure of the piglets’ amygdala was observed by a transmission electron microscope. As shown in Figure 3A,C, the amygdala samples in the control and the music groups exhibited a normal morphology. The ultrastructure of the amygdala in the noise group is shown in Figure 3B. The cytoplasm is darker, the chromatin in the outer nuclear is clustered and unevenly distributed, and several dead cells can be seen.

The expression of Bax, Caspase3, Caspase9, and Bcl2, all of which are related to apoptosis, were analyzed at both the transcription and protein levels. The expression of these genes at the protein level is shown in Figure 3D,E. The expression of Bax, Caspase3, and Caspase9 was higher (*p* = 0.0199, *p* < 0.0001, *p* = 0.0031), while the expression of Bcl2 (*p* < 0.0001) was lower in the noise group than in the control group. The relative expression of Bax (*p* = 0.0144), Caspase3 (*p* = 0.0012), and Caspase9 (*p* < 0.0001) was lower in the music group than in the control group. Meanwhile, the expression of Bax (*p* = 0.0144), Caspase3 (*p* < 0.0001), and Caspase9 (*p* < 0.0001) was higher, while that of Bcl2 (*p* = 0.0016) was lower in the noise group than in the music group. The expression of Bcl2/Bax was lower in the noise group than in both the control (*p* = 0.0082) and music (*p* = 0.0011) groups.

The qPCR results are shown in Figure 3F. The relative transcription of Bax (*p* = 0.0029), Caspase3 (*p* = 0.0003), and Caspase9 (*p* = 0.0004) mRNAs was higher, while the level of Bcl2 (*p* = 0.0108) was lower in the noise group than in the control group. The relative level of Bcl2 (*p* < 0.0001) in the music group was higher than in the control group (*p* = 0.0001), albeit statistically insignificant. Further analyses revealed that, compared with the noise and music groups, the levels of Bax (*p* = 0.0002), Caspase3 (*p* < 0.0001), and Caspase9 (*p* < 0.0001) were higher, while the level of Bcl2 (*p* < 0.0001) was lower. 

### 3.4. Noise-Related Stress Causes Neuroinflammation

**Figure 4:**
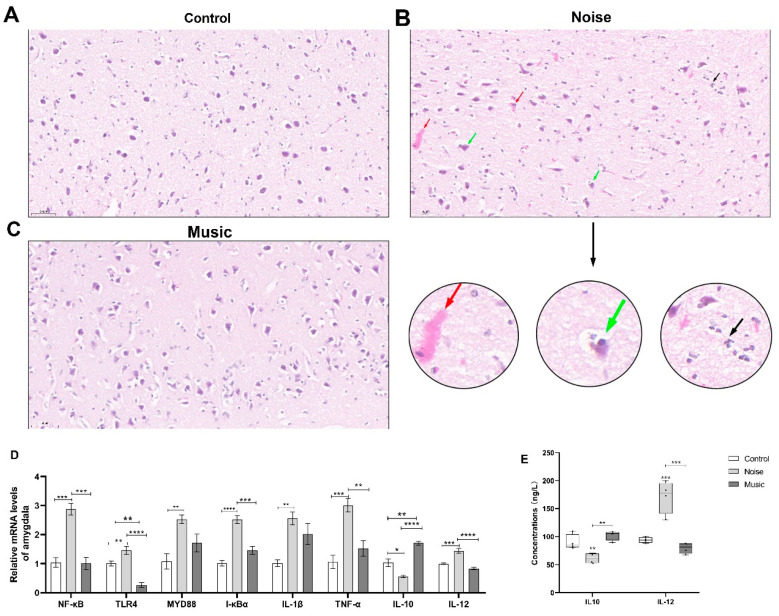
The accumulation of neuroinflammation after treatments. (**A**–**C**) show the H&E staining observations. H&E stained sections of the amygdala (*n* = 4). The amygdala of the control group and the music group displayed normal histology. Hemorrhagic spots (red arrow) with infiltration of inflammation cells (black arrow) can be seen in the noise group; neuronophagia phenomenon (green arrow) appears as well. (**D**) shows the expression of NF-κB-related genes on mRNA levels. (**E**) shows the cytokine levels of the amygdala after treatments. Data are expressed as mean ± SE; significant differences are indicated by asterisks (* *p* < 0.05, ** *p* < 0.01, *** *p* < 0.001, **** *p* < 0.0001; one-way ANOVA, *n* = 4).

Neurons are constantly influenced by external stimuli, and they respond in a manner that maintains the normal functioning of the nervous system. The amygdala ultrastructure is shown in Figure 4A–C. The amygdalae in both the control and music groups displayed a normal histology (Figure 4A,C), but those of piglets in the noise group exhibited hemorrhagic spots with the infiltration of inflammatory cells, and the neuronophagia phenomenon appeared at the same time.

The relative mRNA levels of genes related to the NF-κB pathway in the amygdala are shown in Figure 4D. The relative levels of NF-κB (p56) (*p* = 0.0002), TLR4 (*p* = 0.0344), MYD88 (*p* = 0.0066), IκBα (*p* < 0.0001), IL-1β (*p* = 0.0053), TNF-α (*p* = 0.0008), and IL-12 (*p* = 0.0008) in the noise group were higher than in the control group. In contrast, the level of IL-10 (*p* = 0.0119) was lower in the noise group than in the control group. Meanwhile, the expression of NF-κB (p56) (*p* = 0.0002), TLR4 (*p* < 0.0001), IκBα (*p* = 0.0007), TNF-α (*p* = 0.0046), and IL-12 (*p* < 0.0001) was higher in the noise group than in the music group, while the expression of IL-10 (*p* < 0.0001) was lower in the noise group than in the music group. Compared with the control group, the expression of IL-10 (*p* = 0.0012) was higher, while the expression of TLR4 in the music group was lower (*p* = 0.0017).

The concentrations of IL-10 and IL-12 in the amygdala are shown in Figure 4E. The concentration of IL-10 in the noise group was higher than in both the control (*p* = 0.0097) and music (*p* = 0.0010) groups. Similarly, the concentration of IL-12 in the noise group was higher than in the control (*p* = 0.0005) and music (*p* = 0.0001) groups.

### 3.5. Prolonged Noise Exposure Decreases the Activity of CAT and SOD

**Figure 5:**
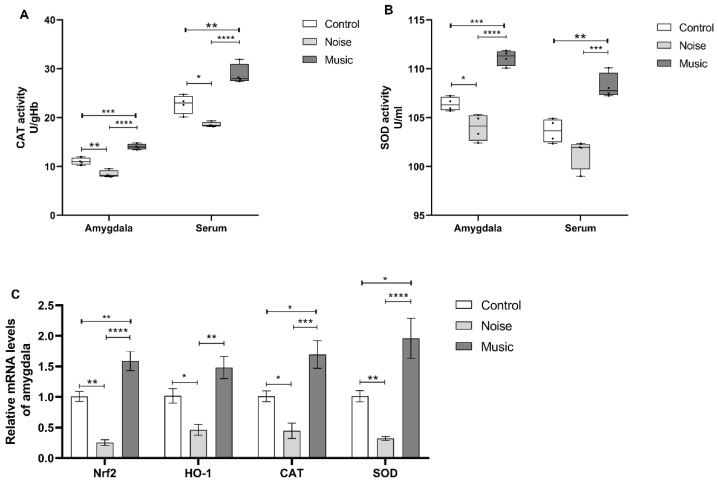
(**A**,**B**) show the activity of antioxidants in amygdala and serum. (**C**) shows the expression of oxidative stress-related genes after treatments. Data are expressed as mean ± SE; data in box-and-whisker graphs are expressed as median +/− quartiles; significant differences are indicated by asterisks (* *p* < 0.05, ** *p* < 0.01, *** *p* < 0.001, **** *p <* 0.0001; one-way ANOVA, *n* = 4).

After pronged exposure to noise, the related stress decreased the activities of CAT and SOD in the amygdala and the serum levels of these enzymes in the piglets (Figure 5A,B). The activity of CAT in the amygdala of the noise group was lower than in the control (*p* = 0.0012) and the music (*p* < 0.0001) groups, but the activity of CAT in the music group was higher than in the control (*p* = 0.0004) group. The activity of CAT was lower in the serum of piglets in the noise group than in the control (*p* = 0.0166) and music (*p* < 0.0001) groups. In the amygdala, the activity of SOD in the noise group was lower than in the control (*p* = 0.0189) and music (*p* < 0.0001) groups, and the activity of SOD in the music group was higher than in the control (*p* = 0.0002) group. Furthermore, the serum SOD activity was lower in the noise group than in the music group (*p* = 0.0001), but no significant difference was observed between the noise and the control groups. The SOD activity in the music group was higher than in the control (*p* = 0.0028) group.

The expression of oxidative stress-related genes in the amygdala at the transcription level is shown in Figure 5C. Here, the relative levels of Nrf2 (noise versus control: *p* = 0.0019; noise versus music: *p* < 0.0001), HO-1 (noise versus control: *p* = 0.0421; noise versus music: *p* = 0.0012), CAT (noise versus control: *p* = 0.0170; noise versus music: *p* = 0.0002), and SOD (noise versus control: *p* = 0.0018; noise versus music: *p* < 0.0001) were lower in the noise group than in both the control and the music groups. At the same time, the levels of Nrf2 (*p* = 0.0098), CAT (*p* = 0.0148), and SOD (*p* = 0.0106) were higher in the control group. 

## 4. Discussion

Many factors, including environmental ones, influence the neural development of infants, which makes their nervous systems highly plastic. In other words, brain development is vulnerable to environmental factors. At infancy, the immune system in the infant brain is weak, and the risk of brain damage caused by external stress is high. Therefore, the early stage of life is a critical period of brain development, and environmental factors play a major role in it.

Animals rely on the functioning of their bodies to fulfill essential requirements such as feeding, movement, and social interaction. Thus, it is necessary to maintain a state of wakefulness. Our results showed that noise exposure increased the activity of piglets and made them stay awake for a longer time. However, the piglets in the noise group showed negative emotion-related behavior over the extended awake period, including manipulative behaviors and aggressive behaviors. Explorative behavior is common in animals in new environments [23]. However, over-explorative behavior in a familiar environment may be related to other factors. A study suggested that pigs redirect their explorative and manipulative behaviors to other penmates [24]. When these explorative or manipulative behaviors involve social contact, they are likely to result in aggressive behaviors, consistent with our results, in which piglets in the noise group displayed more explorative behaviors, manipulative behaviors, and aggressive behaviors. No statistically significant difference was observed in the arousal time between the noise and the music group. Piglets in the music (positive control) group displayed more positive emotion-related behaviors but fewer abnormal behaviors, including aggressive behaviors. According to Russell’s core affect theory [25], the overexpression of explorative behaviors and aggressive behaviors in the noise group may indicate a high arousal degree and negative valence. In other words, noise increased anxiety.

Dopamine is thought to be a “reward” neurotransmitter [23], and the under-secretion of dopamine can lead to anxiety and depression-like behaviors [26,27]. Combined with behavioral performance, it is reasonable to speculate that the dopamine concentration was lower in the noise group but higher in the music group than in both the noise group and control group, since the piglets in the noise group displayed more aggressive behaviors, while the piglets in the music group displayed more positive emotion-related behaviors. However, changes in the concentrations of other neurotransmitters were also observed.

The concentrations of norepinephrine, histamine, taurine, and glutamine were higher in the amygdala after prolonged noise exposure. Noradrenaline, histamine, and taurine have the ability to “arouse” the mind [28,29,30] in a manner similar to the typical excitatory amino acid (glutamic acid). In the present study, the piglets in the noise group were awake longer than those in the other groups. Histidine is the precursor of histamine in the central nervous system (CNS) [31], and histidine de-carboxylation is one of the most important sources of histamine [32]. Histamine synthesis depends on the concentration of histidine [33,34]. In the present study, we found that the concentrations of both histamine and histidine were higher in the serum of piglets in the noise group than in the control group, consistent with other studies. The contribution of taurine to cognitive impairment has been widely studied. Taurine shows a strong time dependence during the process of cognitive development [35]. It is also abundant in the developmental stage, where it serves as a trophic factor for the brain, promoting the proliferation of cells and protecting them from external damage [36,37]. We believe that the higher taurine content in the noise group was due to this protective function against negative stimuli. No statistical difference was observed in the concentrations of GABA and glutamic acid between the music and the control group, indicating the homeostasis of the excitatory and inhibitory neurotransmitters in the piglets. At the same time, the concentration of taurine was higher in the music group than in the control group, which may support the view of the beneficial effect of music on the cognitive development of the amygdala.

It is worth noting that noradrenaline has a “neural gain” effect in the brain, where it amplifies neural communication. Specifically, noradrenaline enhances the activation of excitatory neurons and inhibitory neurons more suppressed [38]. In other words, both noise-induced stress and music increased the concentration of noradrenaline in the amygdala, yet the same results represented different biological meanings, which was reflected in the piglets in the music group exhibiting more positive emotion-related behaviors while the piglets in the noise group mainly exhibited anxiety.

Interestingly, although the piglets in the noise group were more active, the concentrations of GABA and glycine, inhibitory neurotransmitters, were higher. In other words, the reason that the piglets in the noise group remained highly aroused does not seem to be related to the decline in the secretion of inhibitory neurotransmitters. In the process of coping with external pressure, the body is under constant stress, and it resists the effects of negative stimuli by activating certain feedback regulation systems. The feedback regulation system might eliminate the stress caused by external negative stimuli through compensatory mechanisms. For instance, compensatory mechanisms increase the release of neurotransmitters, which could be why the concentration of GABA increased in the amygdala of piglets in the noise group (to reverse their own abnormal secretion). At the same time, however, this type of compensatory increase may bring some unpredictable risks. Maintaining normal physiological concentrations of neurotransmitters is extremely important for homeostasis [39]. The abnormal secretion of neurotransmitters such as glutamic acid could cause neural death through excitotoxicity [40], an unbearable injury. If the clearance of postsynaptic products is ineffective after exposure to external stimuli (noise stress in this study), these uncleared by-products continue accumulating and may result in brain dysfunction and abnormal neuro-behaviors. Apoptosis, neuroinflammation, and oxidative damage may be key steps in these cascades.

In the noise group, the activation of the apoptosis pathway demonstrated that noise-related stress participated in apoptosis in piglets. Apoptosis itself is not passive but rather a planned and programmed death [41,42]. As mentioned earlier, homeostasis of the internal environment is an indispensable condition for cells to perform normal physiological functions. Abnormal apoptosis caused by external stress in the brain may damage the neurons, disrupting their functions. A close relation exists between apoptosis, oxidative damage, and inflammation. In the present study, Nrf2, HO-1, CAT, and SOD expression at the transcription level was lower in the noise group. The decrease in CAT and SOD activity is usually caused by oxidative stress [43], suggesting that prolonged noise causes oxidative damage in the amygdala. Contrarily, the activity of antioxidants was higher in the music group than in the noise and control groups. The mRNA levels of antioxidation-related genes were higher, which further demonstrated that music could be a potential positive stimulus.

The NF-κB signaling pathway plays an important role in immune and inflammatory responses [44,45]. Our results showed that the relative transcription of NF-κB (p65), TLR-4, MyD88, IκBα, IL-1β, and TNF-α mRNAs was higher in the noise group than in the control group, indicating that noise-induced stress activated the expression of NF-κB pathway-related genes. No significant difference was observed in the relative mRNA expression of these genes between the music and the control group, but the expression in both was lower than in the noise group. These findings indicate that music and noise suppress the stimulation of the amygdala cells.

After the activation of the NF-κB pathway, a series of cytokines participate in the proliferation and differentiation of immune cells, immune responses, and pro-inflammatory effects. During the neuroimmune response, the anti-inflammatory cytokines mediate homeostasis to limit excessive inflammation, to prevent undesirable pathological damages [46]. On the one hand, IL-10 is an important neuroimmune cytokine; on the other hand, it is an important anti-inflammatory cytokine that limits antigen presentation to reduce T-cell responses. Thus, in effect, it inhibits the production of pro-inflammatory cytokines. IL-10 is also a neuromodulator that reduces anxiety-like behaviors in mice by inhibiting the release of GABA [46,47], consistent with our findings in which an increase in GABA secretion was observed in the noise group. In the inflammatory response, the expression of the pro-inflammatory cytokine IL-12 is upregulated [48]. IL-12 mainly activates neutrophils, initiating the production of a variety of pro-inflammatory cytokines [49]. Chemokines and cytokines secreted upon the IL-12 activation of immune cells activate the immune system further [50]. In the noise group, the activation of NF-κB pathway-related genes was accompanied by the downregulation of IL-10 and the upregulation of IL-12 production. Combined with the infiltration of numerous inflammatory cells, we speculate that neuroinflammatory accumulation occurred in the amygdala of piglets in the noise group.

The change in the concentrations of inflammatory cytokines and cell metabolic products is not only the strategy that the body utilizes to respond to external stress but may also be the result of the abnormal activation of other pathways. From the above and previous findings, it can be deduced that stress is not a simple challenge to the immune system but an event that requires the cooperation of various systems. As mentioned before, individuals may initiate certain defensive mechanisms against external stress to protect the body. The increase in the taurine concentration, an anti-inflammatory and anti-oxidative damage amino acid, could have been one of these responses in the noise group. However, other factors, including the types and sources of noise and the duration of noise exposure, may affect the actual impact of noise on stress. Thus, further studies are needed to validate our findings.

## 5. Conclusions

In this study, exposure to noise increased the concentration of GABA, which we believe disrupted the metabolism of neurotransmitters produced as a result of noise. The upregulation of the relative mRNA expression of Bax, Caspase3, and Caspase9 and the downregulation of Bcl2 indicated that the activation of the apoptosis pathway was involved in the noise-induced stress process. Moreover, our results showed that noise exposure induced oxidative stress and neuroinflammation. Noise increased the animals’ propensity to engage in aggressive behaviors. In contrast, music promoted the performance of positive emotion-related behaviors, indicating that noise may cause anxiety. Our findings provide practical evidence that music can improve animals’ emotions.

## Figures and Tables

**Table 1:** Ethogram used for behavior observations (2005, Bolhuis).

Behavior		Definition
State behavior	Standing	The body is supported on the ground and the piglets stay in a state of arousal
	Lying	Lying on side or belly
Event behavior	Positive emotion-related behavior	Piglets wagging their tails or playing with penmates, usually including play behavior and tail wagging
	Rooting	Sniffing, touching, or rooting (substrate on floor)
	Pen interaction	Sniffing or biting the trough and grooves with nose or mouth
	Belly nosing	Touching or sniffing any part of the body of a penmate except the head
	Manipulating ears	Nibbling, sucking, or chewing the tail of a penmate
	Manipulating tails	Nibbling, sucking, or chewing the tail of a penmate
	Aggressive behavior	Mutual pushing, biting, ramming, or lifting of penmate

## Data Availability

The data are not publicly available due to privacy.

## Supplementary Materials

The following supporting information can be downloaded at: https://www.mdpi.com/article/10.3390/metabo13080928/s1, Figure S1: Equal-sized pens of each room; Table S1: Primers used for qRT-PCR; Tabel S2 Antibodies used for western blot.

## References

[1] Jarosińska D., Héroux M.-È., Wilkhu P., Creswick J., Verbeek J., Wothge J., Paunović E. (2018). Development of the WHO Environmental Noise Guidelines for the European Region: An Introduction. Int. J. Environ. Res. Public Health.

[2] Münzel T., Schmidt F.P., Steven S., Herzog J., Daiber A., Sørensen M. (2018). Environmental Noise and the Cardiovascular System. J. Am. Coll. Cardiol..

[3] Ma J., Li C., Kwan M.-P., Kou L., Chai Y. (2020). Assessing Personal Noise Exposure and Its Relationship with Mental Health in Beijing Based on Individuals’ Space-Time Behavior. Environ. Int..

[4] Hu B.H., Henderson D., Nicotera T.M. (2006). Extremely Rapid Induction of Outer Hair Cell Apoptosis in the Chinchilla Cochlea Following Exposure to Impulse Noise. Hear. Res..

[5] Hu B.H., Cai Q., Manohar S., Jiang H., Ding D., Coling D.E., Zheng G., Salvi R. (2009). Differential Expression of Apoptosis-Related Genes in the Cochlea of Noise-Exposed Rats. Neuroscience.

[6] Khoshsirat S., Abbaszadeh H.-A., Peyvandi A.A., Heidari F., Peyvandi M., Simani L., Niknazar S. (2021). Apelin-13 Prevents Apoptosis in the Cochlear Tissue of Noise-Exposed Rat via Sirt-1 Regulation. J. Chem. Neuroanat..

[7] Chen F.-Q., Zheng H.-W., Hill K., Sha S.-H. (2012). Traumatic Noise Activates Rho-Family GTPases through Transient Cellular Energy Depletion. J. Neurosci..

[8] Kragel P.A., Čeko M., Theriault J., Chen D., Satpute A.B., Wald L.W., Lindquist M.A., Feldman Barrett L., Wager T.D. (2021). A Human Colliculus-Pulvinar-Amygdala Pathway Encodes Negative Emotion. Neuron.

[9] Tamietto M., de Gelder B. (2010). Neural Bases of the Non-Conscious Perception of Emotional Signals. Nat. Rev. Neurosci..

[10] de Gelder B., van Honk J., Tamietto M. (2011). Emotion in the Brain: Of Low Roads, High Roads and Roads Less Travelled. Nat. Rev. Neurosci..

[11] Corder G., Ahanonu B., Grewe B.F., Wang D., Schnitzer M.J., Scherrer G. (2019). An Amygdalar Neural Ensemble That Encodes the Unpleasantness of Pain. Science.

[12] Tye K.M., Prakash R., Kim S.-Y., Fenno L.E., Grosenick L., Zarabi H., Thompson K.R., Gradinaru V., Ramakrishnan C., Deisseroth K. (2011). Amygdala Circuitry Mediating Reversible and Bidirectional Control of Anxiety. Nature.

[13] Wei P., Liu N., Zhang Z., Liu X., Tang Y., He X., Wu B., Zhou Z., Liu Y., Li J. (2015). Processing of Visually Evoked Innate Fear by a Non-Canonical Thalamic Pathway. Nat. Commun..

[14] Quirk G.J., Armony J.L., LeDoux J.E. (1997). Fear Conditioning Enhances Different Temporal Components of Tone-Evoked Spike Trains in Auditory Cortex and Lateral Amygdala. Neuron.

[15] Sander K., Scheich H. (2001). Auditory Perception of Laughing and Crying Activates Human Amygdala Regardless of Attentional State. Cogn. Brain Res..

[16] Kazi A., Oommen A. (2014). Chronic Noise Stress-Induced Alterations of Glutamate and Gamma-Aminobutyric Acid and Their Metabolism in the Rat Brain. Noise Health.

[17] Cui B., Wu M., She X. (2009). Effects of Chronic Noise Exposure on Spatial Learning and Memory of Rats in Relation to Neurotransmitters and NMDAR2B Alteration in the Hippocampus. J. Occup. Health.

[18] Ravindran R., Devi R.S., Samson J., Senthilvelan M. (2005). Noise-Stress-Induced Brain Neurotransmitter Changes and the Effect of Ocimum Sanctum (Linn) Treatment in Albino Rats. J. Pharmacol. Sci..

[19] Di G., Xu Y. (2017). Influences of Combined Traffic Noise on Anxiety in Mice. Sci. Total Environ..

[20] Guilloteau P., Zabielski R., Hammon H.M., Metges C.C. (2010). Nutritional Programming of Gastrointestinal Tract Development. Is the Pig a Good Model for Man?. Nutr. Res. Rev..

[21] Radlowski E.C., Conrad M.S., Lezmi S., Dilger R.N., Sutton B., Larsen R., Johnson R.W. (2014). A Neonatal Piglet Model for Investigating Brain and Cognitive Development in Small for Gestational Age Human Infants. PLoS ONE.

[22] Li J., Qian Q.H., Zhang R., Liu H., Li X., Bao J. (2020). Effects of Music Stimulus on Behavior Response, Cortisol Level and Immunity Horizontal of Growing Pigs. J. Anim. Sci..

[23] Boissy A., Manteuffel G., Jensen M.B., Moe R.O., Spruijt B., Keeling L.J., Winckler C., Forkman B., Dimitrov I., Langbein J. (2007). Assessment of Positive Emotions in Animals to Improve Their Welfare. Physiol. Behav..

[24] de Jong I.C., Ekkel E.D., van de Burgwal J.A., Lambooij E., Korte S.M., Ruis M.A.W., Koolhaas J.M., Blokhuis H.J. (1998). Effects of Strawbedding on Physiological Responses to Stressors and Behavior in Growing Pigs. Physiol. Behav..

[25] Russell J.A. (2003). Core Affect and the Psychological Construction of Emotion. Psychol. Rev..

[26] Solvi C., Baciadonna L., Chittka L. (2016). Unexpected Rewards Induce Dopamine-Dependent Positive Emotion–like State Changes in Bumblebees. Science.

[27] Bahi A., Dreyer J.-L. (2019). Dopamine Transporter (DAT) Knockdown in the Nucleus Accumbens Improves Anxiety- and Depression-Related Behaviors in Adult Mice. Behav. Brain Res..

[28] Devidze N., Lee A.W., Zhou J., Pfaff D.W. (2006). CNS Arousal Mechanisms Bearing on Sex and Other Biologically Regulated Behaviors. Physiol. Behav..

[29] Tashiro M., Mochizuki H., Iwabuchi K., Sakurada Y., Itoh M., Watanabe T., Yanai K. (2002). Roles of Histamine in Regulation of Arousal and Cognition: Functional Neuroimaging of Histamine H1 Receptors in Human Brain. Life Sci..

[30] Hämmerer D., Hopkins A., Betts M.J., Maaß A., Dolan R.J., Düzel E. (2017). Emotional Arousal and Recognition Memory Are Differentially Reflected in Pupil Diameter Responses during Emotional Memory for Negative Events in Younger and Older Adults. Neurobiol. Aging.

[31] Dai H., Kaneko K., Kato H., Fujii S., Jing Y., Xu A., Sakurai E., Kato M., Okamura N., Kuramasu A. (2007). Selective Cognitive Dysfunction in Mice Lacking Histamine H1 and H2 Receptors. Neurosci. Res..

[32] Adachi N., Liu K., Arai T. (2005). Prevention of Brain Infarction by Postischemic Administration of Histidine in Rats. Brain Res..

[33] Prell G.D., Hough L.B., Khandelwal J., Green J.P. (2002). Lack of a Precursor-Product Relationship Between Histamine and Its Metabolites in Brain After Histidine Loading. J. Neurochem..

[34] Bae O.-N., Majid A. (2013). Role of Histidine/Histamine in Carnosine-Induced Neuroprotection during Ischemic Brain Damage. Brain Res..

[35] Chen C., Xia S., He J., Lu G., Xie Z., Han H. (2019). Roles of Taurine in Cognitive Function of Physiology, Pathologies and Toxication. Life Sci..

[36] Agrawal H.C., Davis J.M., Himwich W.A. (1968). Developmental changes in mouse brain: Weight, water content and free amino acids. J. Neurochem..

[37] Pasantes-Morales H., Hernández-Benítez R. (2010). Taurine and Brain Development: Trophic or Cytoprotective Actions?. Neurochem. Res..

[38] Eldar E., Cohen J.D., Niv Y. (2013). The Effects of Neural Gain on Attention and Learning. Nat. Neurosci..

[39] Win-Shwe T.-T., Mitsushima D., Nakajima D., Ahmed S., Yamamoto S., Tsukahara S., Kakeyama M., Goto S., Fujimaki H. (2007). Toluene Induces Rapid and Reversible Rise of Hippocampal Glutamate and Taurine Neurotransmitter Levels in Mice. Toxicol. Lett..

[40] Choi D.W., Rothman S.M. (1990). The Role of Glutamate Neurotoxicity in Hypoxic-Ischemic Neuronal Death. Annu. Rev. Neurosci..

[41] Niknazar S., Abbaszadeh H.-A., Peyvandi H., Rezaei O., Forooghirad H., Khoshsirat S., Peyvandi A.A. (2019). Protective Effect of [Pyr1]-Apelin-13 on Oxidative Stress-Induced Apoptosis in Hair Cell-like Cells Derived from Bone Marrow Mesenchymal Stem Cells. Eur. J. Pharmacol..

[42] Strasser A., O’Connor L., Dixit V.M. (2000). Apoptosis signaling. Annu. Rev. Biochem..

[43] Li Z., Ali Shah S.W., Zhou Q., Yin X., Teng X. (2021). The Contributions of MiR-25-3p, Oxidative Stress, and Heat Shock Protein in a Complex Mechanism of Autophagy Caused by Pollutant Cadmium in Common Carp (*Cyprinus carpio* L.) Hepatopancreas. Environ. Pollut..

[44] Duncan S.A., Baganizi D.R., Sahu R., Singh S.R., Dennis V.A. (2017). SOCS Proteins as Regulators of Inflammatory Responses Induced by Bacterial Infections: A Review. Front. Microbiol..

[45] Kawasaki T., Kawai T. (2014). Toll-Like Receptor Signaling Pathways. Front. Immunol..

[46] Patel R.R., Wolfe S.A., Bajo M., Abeynaike S., Pahng A., Borgonetti V., D’Ambrosio S., Nikzad R., Edwards S., Paust S. (2021). IL-10 Normalizes Aberrant Amygdala GABA Transmission and Reverses Anxiety-like Behavior and Dependence-Induced Escalation of Alcohol Intake. Prog. Neurobiol..

[47] Suryanarayanan A., Carter J.M., Landin J.D., Morrow A.L., Werner D.F., Spigelman I. (2016). Role of Interleukin-10 (IL-10) in Regulation of GABAergic Transmission and Acute Response to Ethanol. Neuropharmacology.

[48] Chi G., Feng X.-X., Ru Y.-X., Xiong T., Gao Y., Wang H., Luo Z.-L., Mo R., Guo F., He Y.-P. (2018). TLR2/4 Ligand-Amplified Liver Inflammation Promotes Initiation of Autoimmune Hepatitis Due to Sustained IL-6/IL-12/IL-4/IL-25 Expression. Mol. Immunol..

[49] e Habiba U., Rafiq M., Khawar M.B., Nazir B., Haider G., Nazir N. (2022). The Multifaceted Role of IL-12 in Cancer. Adv. Cancer Biol. Metastasis.

[50] Portielje J.E., Gratama J., van Ojik H.H., Stoter G., Kruit W.H. (2003). IL-12: A Promising Adjuvant for Cancer Vaccination. Cancer Immunol. Immunother..

