# Supplementary material for: Effect of Noise and Music on Neurotransmitters in the Amygdala: The Role Auditory Stimuli Play in Emotion Regulation
Source: Metabolites. 2023 Aug 8;13(8):928. doi: 10.3390/metabo13080928 (PMC10456833; doi:10.3390/metabo13080928)

Supplementary Table S1 Primers used for qRT-PCR

Gene	NCBI ID	Primer
Caspase3	NM_214131.1	Forward5'-CCGGAATGGCATGTCGATCT-3'<br>Reverse 5'-AGTCCAATTCTGTGCCTCGG-3'
Bax	XM_003127290.5	Forward 5'-GCCCTTTTGCTTCAGGGTTTC-3'<br>Reverse 5'-CAATGCGCTTGAGACACTCG-3'
BCl2	XM_021099593.1	Forward 5'-AAGCGAGTGGCCCAAGTTTA-3'<br>Reverse 5'-CTGCACTCAGGAGCATGACA-3'
Caspase9	XM_013998997.2	Forward 5'-CAGTGGTGCTGGGGTCTAAG-3'<br>Reverse 5'-GGCCTTGGCAGTCAGGTT-3'
TNF-α	NM_214022.1	Forward 5'-GCACTGAGAGCATGATCCGAGAC-3'<br>Reverse 5'-CGACCAGGAGGAAGGAGAAGAGG-3'
IL-1β	NM_001302388.2	Forward 5'-CAAGCCAGAGAAGCAAGGTGTCC-3'<br>Reverse 5'-GCCGTCCTCAGCAGCAAGAAG-3'
TLR4	NM_001113039.2	Forward 5'-GACGAAGACTGGGTGAGGAATGAAC-3'<br>Reverse 5'-CCTGGATGATGTTAGCAGCGATGG-3'
MyD88	NM_001099923.1	Forward 5'-CTGCGTCTGGTCCATTGCTAGTG-3'<br>Reverse 5'-TTCTGATGGGCACCTGGAGAGAG-3'
IκBα	NM_001005150.1	Forward 5'-TGGTGTCGCTCTTGTTGAAGTGTG-3'<br>Reverse 5'-GCTGCTGTATCCGAGTGCTTGG-3'
NF-κB (p65)	NM_001114281.1	Forward 5'-CTGAGGCTATAACTCGCTTGGTGAC-3'<br>Reverse 5'-CATGTCCGCAATGGAGGAGAAGTC-3'
IL-6	NM_001252429.1	Forward 5'-AAATGTCGAGGCCGTGCAGATTAG-3'<br>Reverse 5'-GGGTGGTGGCTTTGTCTGGATTC-3'
IL-10	NM_214041.1	Forward 5'-CAGCAAGCTCCAGCTCATCCATC-3'<br>Reverse 5'-CAGCAGAAGCAGCAGTGACAGG-3'
IL-12	NM_214097.2	Forward 5'-TCTCCCCCAAATCACATCCAATAA-3'<br>Reverse 5'-TTTCCCGGCTTTGAGTCAGG-3'
Nrf1	XM_021078993.1	Forward 5'-GAAGCTGTCCAGGGGCTTTA-3'<br>Reverse 5'-ATCCATGCTCTGCTACTGGG-3'
HO-1	NM_001004027.1	Forward 5'-TGGCGTCCTTGTACCACATC-3'<br>Reverse 5'-GTCACGGGAGTGGAGTCTTG-3'
CAT	NM_214301.2	Forward 5'-CCTGCAACGTTCTGTAAGGC-3'<br>Reverse 5'-GCTTCATCTGGTCACTGGCT-3'
SOD1	NM_001190422.1	Forward 5'-CAGGGCACCATCTACTTCGAG-3'<br>Reverse 5'-TTACACCACAGGCCAAACGA-3'
β-actin	XM_021086047.1	Forward 5'-GGCACCACACCTTCTACAACGAG-3'<br>Reverse 5'-TCATCTTCTCACGGTTGGCTTTGG-3'

Supplementary Table S2 Antibodies used for western blot

Primary antibodies	Dilution ratio
Bax	1:1000
Caspase3	1:1000
Caspase9	1:1000
BCl2	1:1000
GAPDH	1:1500

Figure S1 Equal-sized pens of each room.

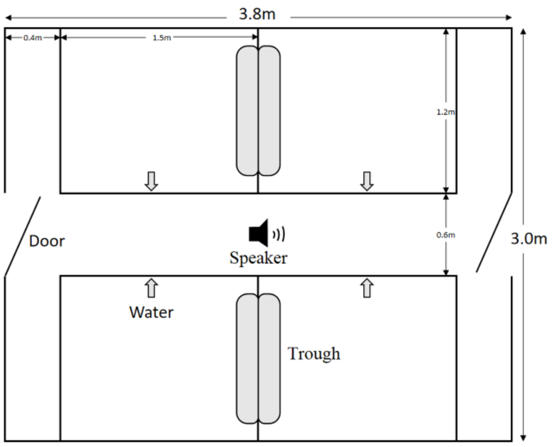

Supplement: Supplementary file 1 [file metabolites-13-00928-s001.zip › Supplementary materials.pdf]
